# A comprehensive approach to risk factors for upper arm morbidities following breast cancer treatment: a prospective study

**DOI:** 10.1186/s12885-021-08891-5

**Published:** 2021-11-20

**Authors:** Ifat Klein, Leonid Kalichman, Noy Chen, Sergio Susmallian

**Affiliations:** 1 Department of Physical Therapy, Assuta Medical Center, Tel Aviv, Israel; 2 Department of physical therapy, Faculty of Health Sciences, Recanati School for Community Health Professions, Ben-Gurion University of the Negev, Beer Sheva, Israel; 3 Department of Surgery, Assuta Medical Center, 20 Habarzel Street, 69710 Tel Aviv, Israel; 4 Faculty of Medicine, Ben Gurion University of the Negev, Beer Sheva, Israel

**Keywords:** Functional limitations, Pain, Physical activity, Physical therapy

## Abstract

**Background:**

Breast cancer surgery frequently causes deficiencies in shoulder functioning. The study pourpode is to identify risk factors for prolonged pain, reduction in function, and decrease in range of motion (ROM) in BC patients.

**Methods:**

A prospective cohort study was designed in a private hospital; between October 2018 and April 2019 with a follow-up of 6 months. Patients following BC surgery, were divided by arm morbidities, and the different risk facrors were evaluated using univariate analysis and logistic regression.

**Results:**

A total of 157 patients were included in the study. Risk factors for functional disabilities included; pain levels during hospitalization NPRS 1.2 (±0.8) compared to patients with no disabilities 0.5 ± 0.7 (*p* = .006), the size of tumors more than 1.4 ± 0.8 cm. compared with no morbidities 0.8 ± 0.9 cm. (*p* = .046), and breast reconstructions (*p* = .030). Risk factors for prolonged pain includes mastectomy (*p* = .006), breast reconstruction (*p* = .011), more than three dissected lymph nodes (*p* = .002), the presence of preoperative pain (*p* < .001), in-hospital pain (*p* < .001), axillary web syndrome (*p* < .001) and lymphedema (*p* < .001). Risk factors for decreased ROM were more than three dissected lymph nodes (*p* = .027), radiation (*p* = .018), and the size of dissected tissue (*p* = .035). Postoperative physical therapy appears to reduce the incidence of prolonged pain (*p* = .013) and regular physical activity may reduce long term functional disabilities (*p* = .021).

**Conclusions:**

Upper arm morbidity following BC treatments affect up to 70% of the patients. Identifying the different risk and beneficial factors may improve awareness among physicians to refer patients to early rehabilitation programs and thus avoid chronic morbidity and improve the course of recovery.

**Trial registration:**

The study was registered in Clinical trial with the ID number: NCT03389204.

**Supplementary Information:**

The online version contains supplementary material available at 10.1186/s12885-021-08891-5.

## Background

Breast cancer (BC) surgeries and treatments can cause arm morbidity that can progress beyond 2.5 years [1]. The most common complaints after breast surgery are postoperative pain reported up to 68% of patients [2], functional limitations in up to 59% of patients after mastectomy and quadrantectomy [3], and decreased range of motion (ROM) in 24–53% [4]. Postoperatively axillary web syndrome (AWS) may cause pain and movement limitations in up to 68% of patients [5]. Lymphedema reported in a prevalence of 6–52% especially after axillary lymph node dissection (ALND), cause functional limitations which are worsening by adjuvant treatments and tissue damage [6].

Risk factors for prolonged pain, functional and movement disabilities, can be varied including factors associated with the type and extent of surgery, as mastectomy and breast reconstructive surgeries cause more pain and limitations than lumpectomies [7]. Non-surgical oncologic treatments also affect the course of recovery, neoadjuvant chemotherapy may result in a reduction in grip strength, shoulder abduction and flexion ROM [4]. Whereas, radiation therapy increases the odds of lymphedema and shoulder restriction compared with non-irradiated patients [8]. Tumor characteristics such as Lymphovascular invasion is additional risk indicator for lymphedema [9]. Furthermore, personal factors can include age, as women under 50 years are susceptible to develop chronic pain [10], obesity that causes a higher incidence of lymphedema [11], history of previous BC treatments might increase the possibility of morbidity and adversely affects recovery [12]. Other factors that need to be considered and can affect the course of recovery are psychological, emotional state [13] and even posture [14].

Upper arm morbidity significantly affects BC survivors’ ability to return to work [15], to full movement, to physical activity [16], and causes a long-term decline in quality of life [17]. The prevailing approach today is preventive intervention and follow-up to identify arm morbidity and lymphedema, therefore, identifying risk factors can help caregivers identify high-risk patients for the morbidity, and to provide treatment according to the patient’s needs [18]. The comprehensive rehabilitation approach after BC treatments and surgeries, addresses various aspects of morbidity, including pain, edema, function, flowing movement and strength of the upper extremity [19], in order to return the patients to full function as they did before the diagnosis.

The treatment is usually combined according to complex decongestant therapy, from components of physical activity, adapted exercises, and for lymphatic patients elastic bandaging and lymphatic massage [20, 21].

The aim of this study is to identify risk factors affecting the recovery of the arm in BC survivors, enabling a personalized treatment plan for patients at risk for arm morbidity**.**

## Methods

A prospective trial was conducted in a single medical center in women that underwent BC surgery between October 2018 and April 2019. The trial was approved by the Institutional Helsinki Review Board- Assuta Medical Center (approval number: 0122–17 ASMC) and was registered on the National Institutes of Health’s website (ClinicalTrials.gov; study identifier NCT03389204). All patients provided written informed consent before enrollment.

### Patients

Inclusion criteria: functionally independent women ≥18 years old with a diagnosis of BC, and planned surgical intervention. Exclusion criteria were: benign disease of the breast, cognitive disorders, fibromyalgia or chronic pain disorders, neurological disorders causing permanent disability, previous breast surgery, lymphedema before the surgery, previous shoulder surgery or injuries causing limited ROM, severe systemic diseases.

### Study design

Patients were evaluated for the presence of functional disabilities, chronic pain, flexion, and abduction ROM, before and 6 months after treatment. Patients were divided into two groups, one that includes patients with upper arm morbidity, disabilities or pain at 6 months follow-up and the second group of patients without any arm morbidity. The different risk factors for arm morbidity included personal factors; age, body mass index (BMI), factors related to the type of surgical procedure performed, such as; the size and stage of the tumor, number of lymph nodes dissected, in addition to the effect of the different oncologic treatment administrated.

The data collection and analysis were conducted by a single investigator and therefore without blindness.

### Outcome measures

Primary outcome measures- The QuickDASH instrument [22] (disabilities of the arm, shoulder, and hand) a short 11-items questionnaire was used for assessing the physical function and symptoms of the upper limbs. Functional limitations were defined as QuickDASH values of 16 and higher (based on the minimal clinically important difference of 16 points [23]). Abduction ROM was evaluated using DrGoniometer application [24]. Limitations in ROM were defined as a range lower than 156 degrees (180 minus the minimum clinical difference which is 24 degrees [25]. The average pain levels were evaluated using the Numeric Pain Rating Scale (NPRS) [26], pain levels were rated from 0 (no pain) to 10 (worst pain). Pain levels were calculated on average over 24 h, before surgery, at the time of hospitalization, and 6 months after surgery. Prolonged pain was defined as the presence of pain (higher than 0 by NPRS) at 6 months.

Secondary outcome measures- Lymphedema and the presence of AWS were examined using a self-reported questionnaire (according to a diagnosis made by a doctor/ physical therapist). Risk factors from the surgical report, the pathological report, oncological treatments and pain levels during hospitalization were taken from the medical record.

### Statistical analysis

Statistical analysis was performed using the SPSS statistical package, Version 21 (SPSS Inc., Chicago, IL, USA). The significance level was set at *p* < .05.

Sample size estimation was calculated using the PS Power and Sample Size Calculations software (Version 3.0, January 2009). QuickDASH instrument was the main outcome measure used to evaluated shoulder functional disability. QuickDASH score after BC surgery [27] (43.2 ± 18) were compared with the general population [28] (10.1 ± 14.6), using this data and the probability of type I error was 0.05, and the probability of type II error was 0.2, 30 experimental subjects and 30 control subjects were needed to reject the null hypothesis. Since six different sub-groups were evaluated, 180 patients were planned to enter the study.

The non-parametric Mann-Whitney rank-sum test for independent samples was applied for testing the statistical significance of the difference between continuous parameters (age, BMI, tumor size, pain, ROM, function values, etc.). Nominal variables were evaluated by the chi-squared test; cancer grade, physical activity, job status, family status, type of surgery, type of oncological treatment, presence of lymphedema and AWS. Logistic regression was used in three different models to evaluate associations between the three outcome measures and a set of covariate variables as risk and beneficial factors. The three regression models examined the adjusted odds ratios (ORs) with confidence intervals (CIs) of 95%.

## Results

### Patients

A total of 188 patients were recruited to the study, of them 28 were excluded; one (3.6%) had a nonmalignant tumor, 17 women (60.7%) had previous breast surgery, five (17.9%) had limited shoulder ROM mostly rotator cuff tears, two (7.1%) had lymphedema, and three (11%) had fibromyalgia. Of 160 remaining patients, three did not complete the follow-up and therefore only 157 patients entered the study cohort.

**Fig. 1:**
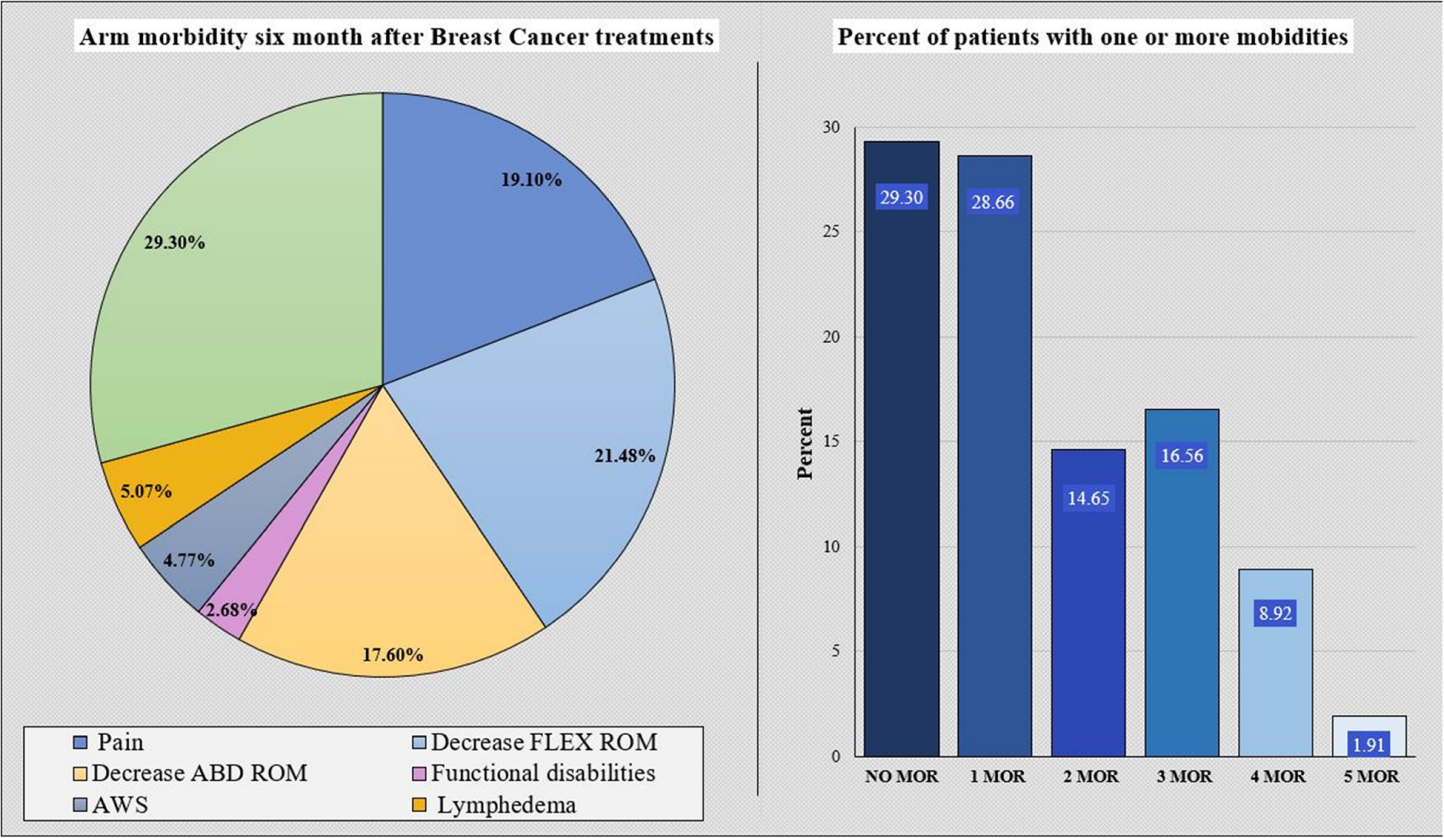
Arm morbidity six-month postoperative after breast cancer treatments. A 29.3% of the patients did not present morbidity at 6 months of follow-up, 70.7% still had some type of morbidity, among the most common: pain and ROM restriction, it was also possible to verify the existence of more than one morbidity in more than 42% of the participants. *Abbreviations*: ROM- Range of motion, Flex- Flexion, Abd- abduction, AWS- Axillary web syndrome, MOR- Morbidities

The patients mean age was 52.2 ± 12.9, with a mean BMI of 25.0 ± 4.4. Patients in both groups, with and without arm morbidity, were similar in values of age, BMI, cancer grade, level of physical activity family status, and type of job (Table 1). Six months postoperatively 111 (70.70%) patients had reported having arm morbidities including pain in, reduced function, diminished ROM, Lymphedema and AWS. The relative percentages of patients with arm morbidities at 6 months; among the subjects who reported morbidity, more than 42% of them reported more than one morbidity as seen in Fig. 1 and Supplementary Table S6.

**Table 1:** Descriptive statistics of the study sample by arm morbidity

Variable	Morbidities *N* = 111	No-morbidities *N* = 46	*p*-value
Age (years/SD)	52.2 ± 12.4	52.1 ± 14.2	.998
BMI (kg/m^2^/SD)	25.9 ± 4.3	24.9 ± 4.7	.634
Hospital stay (days/SD)	1.6 ± 0.8	1.4 ± 0.6	.139
right Dominance side (N/%)	94 (84.7)	41 (89.1)	.465
*Cancer grade:*			
Non (N/%)	28 (25.2)	20 (43.5)	.108
Low grade (N/%)	23 (20.7)	7 (15.2)	
Moderate grade (N/%)	35 (31.5)	10 (21.7)	
High grade (N/%)	25 (22.5)	9 (19.6)	
*Physical activity:*			
No PA (N/%)	38 (34.2)	12 (26.1)	.696
Light PA (N/%)	29 (26.1)	14 (30.4)	
Moderate PA (N/%)	30 (27.0)	12 (26.1)	
Vigorous PA (N/%)	14 (12.6)	8 (17.4)	
*Job-status:*			
Not working (N/%)	38 (34.2)	19 (41.3)	.696
Part-time (N/%)	20 (18.0)	7 (15.2)	
Full time (N/%)	53 (47.7)	20 (43.5)	
*Family status:*			
Single (N/%)	10 (9.1)	7 (15.2)	.477
Married (N/%)	83 (74.5)	35 (76.1)	
Divorce (N/%)	13 (11.8)	3 (6.5)	
Widow (N/%)	5 (4.5)	1 (2.2)	
H. phys. Therapy (N/%)	48 (43.2)	24 (52.2)	.308

The table describes the number of women who reported any arm morbidities (out of 4 diseases; decreased function, pain, or limitation in flexion or abduction ranges). Categorical variables are presented as number and percentage and continuous variables are presented as mean and standard deviation **(SD)**. *Significant *p*-value (< 0.05)

*Abbreviations: BMI* Body mass index, *ROM* range of motion, *SLNB* sentinel lymph node biopsy, *ALND* axillary lymph node dissection. *H. phys. Therapy* received physical therapy during a hospital stay

### Risk factors for functional disabilities

Seven participants out of 157 lacked data and were therefore not included in the analysis. Risk factors for functional disabilities of the upper arm were found to be attributed to the tumor size, as patients with arm morbidity had bigger tumors 1.4 ± 0.8 compared to patients with no morbidities, 0.8 ± 0.9 (*p* = .046). A higher percentage of patients after breast reconstructions had reported decreased function, nine (55.6%) compared with patients that did not undergo reconstructions 141 (33.3%), *p* = .030. In addition, patients with function disabilities 6 months postoperatively had reported higher pain levels during hospitalization, 1.2 ± 0.8, compared to patients with no disabilities, 0.5 ± 0.7 (*p* = .006), shown in Table 2 and Supplementary Table S7.

**Table 2:** Univariate results of potential risk and beneficial factors for functional disabilities, prolonged pain, and decreased abduction and flexion ROM, 6 months postoperatively

Variable	Functional disabilities			Prolonged pain			Decrease ROM		
	Yes *n* = 9	No *n* = 141	*p*- value	Yes *n* = 64	No *n* = 83	*p*- value	Yes *n* = 59	No *n* = 95	*p*-value
**Risk factors**									
Tum. size (cm)	1.4	0.8	.046*	1.0	0.9	.880	0.9	0.9	.359
Tissue excised (cc^3^)	404.0	341.0	.372	487.8	317.6	.090	520.6	389.6	.035*
Mastectomy	55.6%	32.6%	.159	46.9%	25.3%	.006*	42.2%	30.5%	.134
Reconstruction	55.6%	33.3%	.030*	45.3%	25.3%	.011*	39.0%	31.6%	.347
Lymph node no.	3.2	2.9	.115	3.7	2.4	.002*	3.4	2.6	.027*
Hospital pain	1.2	0.5	.006*	0.9	0.3	< .001*	0.7	0.5	.078
Daily drain (cc)	31.1	20.1	.268	32.2	12.7	< .001*	23.3	19.9	.364
Neoadjuvant T	11.1%	22.0%	.440	28.1%	16.9%	.101	27.1%	18.9%	.235
Adjuvant T	33.3%	37.6%	.798	46.9%	32.5%	.077	42.4%	35.8%	.414
Radiation T	88.9%	59.6%	.080	62.5%	60.2%	.780	72.9%	53.7%	.018*
IORT	11.1%	7.9%	.733	3.2%	9.6%	.132	3.5%	10.5%	.120
Pre-OP function	7.6	3.5	.617	3.8	3.2	.637	4.7	3.1	.720
Pre-OP pain	0.3	0.2	.272	0.3	0.01	.025*	0.2	0.1	.214
Lymphedema	22.2%	9.2%	.210	21.9%	1.2%	< .001*	13.6%	8.0%	.211
AWS	0	10.9%	.297	20.6%	3.6%	< .001*	10.3%	10.8%	.937
**Beneficial factors**									
Physical activity	33.3%	70.3%	.021*	62.5%	73.5%	.154	61.1%	74.1%	.081
Physical therapy	22.2%	47.4%	.143	35.9%	56.6%	.013*	41.7%	49.4%	.332

The table describes morbidity of the upper limb 6 months after breast surgery. Functional limitation (using QuickDASH), pain (using numeric pain rating scale), and reduction in the abduction ranges of motion, are presented as “yes” meaning exist or “no” in each of the risk factors. Categorical variables are presented as number and percentage and continuous variables are presented as mean and standard deviation **(SD)**. *Significant *p*-value (< 0.05)

*Abbreviations*: *Tum.* tumor, *ROM* range of motion, *T.* treatment, *IORT* intraoperative radiation therapy, *Pre-OP* Preoperative, *AWS* Axillary web syndrome

### Risk factor for prolonged pain

Ten patients had missing data and therefore not included in this analysis. The risk factors included mastectomy procedure in (46.9%) patients compared to lumpectomy (25.3%), *p* = .006. Patients after breast reconstruction reported prolonged pain in higher percentage (45.3%) compared with patients without pain (25.3%), *p* = .011. In addition, patients that had reported prolong pain had a higher number of dissected lymph nodes 3.7 ± 3.0, compared to patients that did not report pain, 6 months postoperatively 2.4 ± 2.7 (*p* = .002) (Supplementary Table S8). The presence of preoperative pain, as higher preoperative pain values were reported in patients with prolonged pain 0.3 ± 0.9, compared to non-painful ones 0.01 ± 0.4 (*p* = .026) (Supplementary Table S9). Likewise, higher pain levels during hospitalization can affect prolonged pain 0.9 ± 1.0 compared to non-painful patients, 0.3 ± 0.5 (*p* < .001). The two common side effects of surgery and treatments are lymphedema and AWS, when patients who suffered from these pathologies, reported prolonged pain in higher percentages compared to patients without these complications, *p* < .001 and *p* = .001, respectively.

### Risk for decreased ROM

Two participants were not included in this analysis due to missing data. Relevant decreased ROM was contributed to a higher number of lymph nodes dissected 3.4 ± 2.8 compared to patients without movement limitation 2.6 ± 2.9 (*p* = .027). Radiation therapy resulted in a greater decrease in ROM 72.9%, compared with women who were not treated with radiation 53.7% (*p* = .018). The size of dissected tissue may be an additional factor as patients with decrease ROM underwent a bigger dissection 520.6 ± 662.7 compared to women without decrease ROM 389.6 ± 449.4 (*p* = .035).

No significant associations were found between the three arm morbidities examined in this study for; age, BMI, chemotherapy and intraoperative radiotherapy (Supplementary Table S10 and Supplementary Table S11).

### Beneficial factors

Physical active patients had less functional disabilities, as only (33.3%) of the patients who reported functional impairment exercised routinely compared with (70.3%) women who had no functional impairment (*p* = .021).

Providing physical therapy treatment during hospitalization and after discharge, might have a beneficial effect on arm function and pain, as from the painful women 64 (35.9%) received physical therapy treatments compared to 83 (56.6%) patients who receive treatment and had no pain (*p* = .013).

### Logistic regression

When we examined the three models in logistic regression very few factors were found to be significant in predicting risk for upper arm morbidity (Table 3). Furthermore, the models were more comprehensive when factors that improved recovery were introduced such as exercising regularly and receiving postoperative physical therapy.

**Table 3:** Logistic regression models of potential risk factors and beneficial factors, 6 months postoperatively, according to the three arm morbidities evaluated in the study

Variable		Model 1 Functional disabilities		Model 2 Prolonged pain		Model 3 Decreased ROM	
		OR (95% CI)	*P*- value	OR (95% CI)	*P*- value	OR (95% CI)	*P*-value
Risk Factors	Age	1.07 (0.96–1.19)	.852	1.00 (0.96–1.64)	.180	1.01 (0.98–1.05)	.367
	BMI	0.94 (0.76–1.14)	.545	0.99 (0.89–1.16)	.885	0.98 (0.89–1.08)	.737
	Tumor Size	0.88 (0.55–1.66)	.623	0.98 (0.59–1.64)	.986	0.88 (0.55–1.39)	.595
	In-hospital pain	3.19 (0.83–12.04)	.089	2.46 (1.19–5.08)	.014*	1.05 (0.61–1.80)	.850
	Mastectomy	2.71 (0.11–64.90)	.299	2.15 (0.75–6.12)	.152	1.82 (0.23–13.95)	.564
	Br. Recons.	7.87 (0.9–68.7)	.062	0.83 (0.96–7.27)	.871	1.35 (0.17–10.37)	.665
	Lymph Node	0.85 (0.20–3.58)	.834	1.14 (0.97–1.33)	.097	1.08 (0.92–1.27	.298
	Lymphedema	11.14 (0.17–36.38)	.058	33.18 (3.27–336.0)	.003*	6.45 (1.04–40.29)	.045*
	AWS	11.02 (0.92–131.61)	.233	14.03 (2.54–77.36)	.002*	0.93 (0.20–4.29)	.934
	Neoadj. Treat.	0.25 (0.03–2.10_	.205	1.58 (0.32–7.76)	.571	0.67 (0.20–3.36)	.792
	Adj. Treat.	0.32 (0.02–3.89)	.375	0.93 (0.38–2.26)	.889	1.62 (0.55–4.72)	.372
	Radiotherapy	7.02 (0.54–90.21)	.1.34	0.93 (0.28–3.03)	.906	3.39 (1.24–9.21)	.017*
	6 m. Function	–	–	1.20 (1.07–1.34)	.001*	0.91 (0.84–0.99)	.030*
	Preop. Pain	0.13 (0.01–0.26)	.005*	1.97 (0.95–4.10)	.067	0.90 (.562–1.34)	.612
	Preop. function	0.06 (0.12–0.38)	.002*	0.36 (0.08–1.42	.160	0.20 (0.63–0.69)	.011*
	Preop. ROM	1.03 (0.96–1.11)	.343	0.99 (0.94–1.04)	.815	0.87 (0.81–0.93)	< .001*
Beneficial Factors	H. PT.	0.00 (0.01–0.97)	.047*	0.13 (0.04–0.38)	< .001*	0.26 (0.10–0.70)	.008*
	PA	0.34 (0.09–1.20	.094	0.41 (0.04–0.33)	.105	0.67 (0.43–1.05)	.082
		Nagelkerke R^2^ 0.621		Nagelkerke R^2^ 0.619		Nagelkerke R^2^ 0.449	

*Abbreviations*: *BMI* Body Mass Index, *Preop.* preoperative, *Br. Reccons.* Breast Reconstruction, *AWS* Axillar Web Syndrome, *Neoadj. Treat.* Neoadjuvant Treatment, *adj. Treat.* Adjuvant Treatment, *6 m.* 6 months, *ROM* Range of Motion, *H. PT.* Hospital Physical Therapy, *Ph. Act.* Physical Activities

*Significan *p*- value .005

The risk factors found for functional disabilities were preoperative pain (OR 0.13, CI 0.01–0.26, *p* = .005) and preoperative function (OR 0.06, CI 0.12–0.38, *p* = .002). Moreover, physical therapy may help reduce disability (OR 0.00, CI 0.01–0.97, *p* = .047).

The risk factors found for prolonged pain were in-hospital pain higher than NPRS 1 (OR 2.46, CI 1.19–5.08, *p* = .014), decreased function at 6 months (OR 1.20, CI 1.07–1.34, *p* = .001), lymphedema (OR 33.18, CI 3.27–336.0, *p* = .003) and AWS (OR 14.03, CI 2.54–77.36, *p* = .002). Physical therapy may be beneficial in reduction of prolonged pain incidences (OR 0.13, CI 0.04–0.38, *p* < .001).

Risk factors found for decreased ROM were lymphedema (OR 6.45, CI 1.04–40.29, *p* = .045) radiation therapy (OR 3.39, CI 1.24–9.21, *p* = .017), preoperative decreased ROM (OR 0.87, CI 0.81–0.93, *p* < .001), preoperative decreased function (OR 0.20, CI 0.63–0.69, *p* = .011) and at 6 months (OR 0.91, CI 0.84–0.99, *p* = .030). Physical therapy as a beneficial factor (OR 0.26, CI 0.10–0.70, *p* = .008). The different risk factors are summarized in Table 4.

**Table 4:** A summary table of the risk factors found to be significant for arm morbidity 6 months after surgery

Risk factors	Functional disability	Prolonged pain	↓ROM
In-hospital pain > 0.5	**√**	**√**	
Radiation therapy			**√**
Lymph nodes dissected > 3		**√**	**√**
Breast reconstruction	**√**	**√**	
Tumour size > 1	**√**		
Tissue size			**√**
Pre-OP pain > 1		**√**	
Daily drain fluid > 20 cc		**√**	
Lymphedema		**√**	
AWS		**√**	
Mastectomy		**√**	

*Abbreviations*: *Funct. Disab.* Functional disabilities, *Pr.* prolonged, *↓* Decreased, *Pr-OP.* Preoperative, *AWS* axillar web syndrome

## Discussion

BC treatments are a common cause for prolonged arm morbidity [4]. Since lymphedema has been studied in great depth in literature, this study examines the other long term arm morbidities that affect the lives of women recovering from BC, in a broad view.

Six months after surgery, the most common complaint reported by 40% of patients was the pain. The results of this study found, like many studies before us, that the extension of surgery has a role in predicting prolonged pain, as mastectomy surgeries cause more pain than lumpectomy [2, 29]. Nevertheless, contrary to expectations, when examining whether a relationship between the amount of tissue removed and the risk of developing prolonged pain exists, we found no significance.

From this cohort, it was found that during hospitalization, even very low pain levels i.e., 0.5 NPRS and above, can affect long-term recovery. Similar results were found in several studies [30–32], demonstrating that the severity of acute postoperative pain and inadequate pain management were associated with an increased likelihood of persistent pain, although the reported pain levels were higher than in our cohort of patients. Nevertheless, in the case of acute pain, it is difficult to isolate the causes, since higher pain scores during hospitalization may be caused by larger surgeries, axillary drains and emotional factors [33]. Legeby et al. found similar results, as patients undergoing extensive surgeries such as immediate breast reconstructions and ALND were at a higher risk for increased pain during hospitalization, and found it to be a predictor of chronic pain [34]. There is an agreement in the literature regarding the role of ALND as a risk factor for chronic pain [2], as well as for the development of lymphedema [35]. Lymphedema was found in this study consistently to other studies, as an additional risk factor for chronic pain [36, 37]. Moreover, the results of this study indicates that even the dissection of 3–4 lymph nodes, has an adverse effect on prolonged pain and decreased ROM, compared to the removal of only 1–2 lymph nodes. Hack et al., demonstrate that the greater the number of lymph node dissected greater the morbidities, including infections, restriction of movements lymphedema and more [38], while, our study analyses all types of oncological treatment of BC and not confined only to surgery.

Miaskowski et al. found that the presence and number of surgical drains placed in the armpit or breast cause moderate pain 6 months after the operation [31]. Our results adds that more than 20 cc of accumulated daily drain secretions increases the risk of prolonged pain.

Moreover, in concurrent to prior literature [2, 4, 39] it was found that preoperative pain may be another important risk factor for prolonged pain, nonetheless, there is difficulty in determining the cause, as there may be other related factors such as neoadjuvant chemotherapy or the tumor itself [40]. AWS was found significantly associated with prolonged pain, comparable to previous reports, finding that the tendon that extends from the armpit toward the arm causes pain and limitation in function [5].

Unlike previous studies, some of which reported young age as a risk factor for prolonged pain and some reported older age [2, 33, 36], we didn’t find association between age and prolonged pain. Similar results were obtained regarding BMI, which contrasting to what was reported in the previous literature [2, 41], was not found to be a risk factor of prolonged pain.

In addition, no associations were found in this cohort between oncology treatments and long-term arm morbidity including pain, decreased function, or ROM, unlike previous studies, which found that radiation therapy [2, 42] and chemotherapy [32, 35] as predictive factors.

A decrease in ROM is the second common complaint in the sample, reported by a third of the participants. Three risk factors were found to be associated with decreased ranges including radiation therapy, which was previously found to causes prolonged pain [2, 40]. Nevertheless, regarding the effect of radiation on ROM reduction, there is conflicting evidence, while some authors demonstrated an associations [43, 44], others did not find significant results [38, 45]. This study results did find radiation to have an adverse effect on shoulder ROM. The number of lymph nodes removed was found to be associated with a decrease in ROM, the results suggest that even removal of 3–4 lymph nodes may result in movement restrictions. Our results examining in more depth, the results of previous researchers such as Kootstra how found that 7 years after BC surgery, 40% of the women after SLNB and 70% out of women after ALND had arm impairments [29]. The third factor that might affect ROM, is the size of breast tissue dissected, as the mastectomy procedure of large breast tissue was found in our results to be a potential cause of limitation in ROM. Many studies in the past, have found that extensive surgeries adversely affect ROM, relative to conservative surgeries [46], nonetheless to the best of our knowledge the amount of tissue removed has not been examined. In addition, contrary to previous reports, we did not find any significant associations between neoadjuvant treatments and a decrease in ROM [4].

The results of our analysis suggest that pain higher than NPRS 0.5 during hospitalization is a potential risk factor not only for prolonged pain but also for long-term disability. The association between pain and function disability were previously explored by Bosompra et al. who found that pain intensity and swelling of the arm are related to functional disability [47], nevertheless to the best of our knowledge the potential association of functional decline to pain during hospitalization was examined in this study for the first time.

In concurrent with previous literature that demonstrated that breast reconstruction surgery, whether it is a tissue expander, implant-based reconstruction, or autologous reconstruction causes functional limitation [48], our data that evaluated mainly implant-based reconstruction suggests that in a small percentage of patients, the effect will last for more than 6 months.

The size of the tumor removed might be another risk factor, as in our sample, patients that had tumors larger than 1 cm reported more functional disabilities, and to the best of our knowledge, no previous studies have reported a similar association.

Two beneficial factors were examined; postoperative physical therapy treatment and consistent physical activity. Previous studies found that postoperative physical therapy is effective in improving functional use of the affected arm [22, 49], our results suggest that in addition, postoperative physical therapy may reduce the incidence of prolonged pain.

The benefits of physical activity were explored in depth in various aspects of research, finding multiple benefits for women with BC, which include a reduction in mortality, in the recurrence of the disease to the relief of the symptoms of oncological treatments [50, 51]. Our results consistent with pervious authors [52], demonstrating that physical active BC patients are less likely to have functional limitations.

Once risk factors for any arm morbidity have been identified for prolonged pain, ROM and function decline, patients with risk factors can be used to pre-identified, and offered a comprehensive rehabilitation approach, which includes early start of physical therapy and physical activity can improve fatigue during chemotherapy [53], improve ROM, quality of life, muscle strength and arm function [54, 55]. Therefore services such as the chemotherapy 54 prospective surveillance care model, that offers long-term multidisciplinary follow-up and treatments tailored to each morbidity, are necessary to provide optimal service for those coping with BC [56].

### Limitations and strengths

The limitations of the study are in the nature of a prospective cohort, which may indicate connections but cannot determine the extent of the influence of the various factors. For this purpose, randomized controlled research is needed, with a longer follow-up can shed definitive results.

In addition, the self-report method of diagnosis in lymphedema and AWS (chosen because of the geographical distance), instead of volume measurement and clinical diagnosis might not provide the precise prevalence of these diseases.

The strengths of the study are in the broad examination of different risk factors that may affect recovery and, in an attempt, to bind the risk factors into models that were intended to predict risk for each morbidity separately (prolonged pain, functional limitation, and limitation in ROM).

## Conclusions

The morbidity of the arm 6 months after BC treatments affects up to 70% of the patients. In-hospital pain, breast reconstructions, and tumour size were correlated with long-term functional disabilities. Mastectomy, the number of lymph nodes removed, pain values in hospital, amount of drain secretion, preoperative pain, lymphedema, and AWS were found to be correlated with prolonged pain. Radiation therapy, the number of lymph nodes removed, and the size of the tissue were found to be correlated with a decrease in ROM. Furthermore, physical therapy may reduce incidence of prolonged pain and physical activity may reduce long term functional disabilities. Consequently, identifying risk factors with an early start of comprehensive physical therapy rehabilitation programs can improve the recovery process of BC patients.

## Supplementary Information

**Additional file 1: Table 5.** Risk factors of any arm morbidities using Mann Whitney and chi-squared test.

**Additional file 2: Table 6.** Crosstab and OR divide by two age groups.

**Additional file 3: Table 7.** Crosstab and OR divide by two BMI groups.

**Additional file 4: Table 8.** Crosstab and OR divide by the mean amount in the drains.

**Additional file 5: Table 9.** Crosstab and OR divide by the size of the tumor.

**Additional file 6: Table 10.** Crosstab and OR divide by the number of dissected lymph nodes.

**Additional file 7: Table 11.** Crosstab and OR divide by the mean pain reported during hospitalization.

**Additional file 8.**

## Data Availability

The data has been deposited in the submission system of the Journal. The datasets used and/or analysed during the current study will be available from the corresponding authors upon reasonable request.

## Abbreviations

BC: Breast cancer
ROM: Range of motion
NPRS: Numeric pain rating scale
CM: Centimetres
AWS: Axillary web syndrome
SLNB: Sentinel lymph node biopsy
ALND: Axillary lymph node dissection
ASMC: Assuta Medical Center
NCT: National clinical trial
BMI: Body mass index
DASH: Disabilities of the arm, shoulder, and hand
OR: Odd ratio
CI: Confidence interval
