# Supplementary material for: A comprehensive approach to risk factors for upper arm morbidities following breast cancer treatment: a prospective study
Source: BMC Cancer. 2021 Nov 20;21:1251. doi: 10.1186/s12885-021-08891-5 (PMC8605604; doi:10.1186/s12885-021-08891-5)
Supplement: Supplementary file 1 — Additional file 1: Table 5. Risk factors of any arm morbidities using Mann Whitney and chi-squared test. [file 12885_2021_8891_MOESM1_ESM.docx]

**Table 5.** Risk factors of any arm morbidities using Mann Whitney and chi-squared test.

| p-value | No arm morbidities  N=46 | Arm morbidity  N=111 | Variable |
| --- | --- | --- | --- |
| 0.413 | 0.3±0.8 | 0.9±0.9 | Tumor size (cm), mean±SD |
| 0.188 | 279.8±394.9 | 384.5±476.4 | Tissue size (cc^3^), mean±SD |
| 0.130 | 12 (26.1) | 43 (38.7) | Mastectomy, n (%) |
| 0.252 | 13 (28.3) | 42 (37.8) | Reconstruction, n (%) |
| 0.008* | 2.1±2.0 | 3.2±3.1 | Lymph node no., mean±SD |
| 0.061 | 0.3±0.4 | 0.7±0.9 | Hospital stay pain, mean±SD |
| 0.058 | 12.0±22.8 | 24.4±36.9 | Daily drain fluids (cc), mean±SD |
| 0.915 | 10 (21.7) | 25 (22.5) | Neoadjuvant T., n (%) |
| 0.608 | 19 (41.3) | 41 (36.9) | Adjuvant T., n (%) |
| 0.261 | 25 (54.3) | 71 (64.0) | Radiation T., n (%) |
| 0.773 | 4 (8.7) | 8 (7.2) | IORT, n (%) |
| 0.105 | 4.4±6.6 | 3.4±6.8 | Pre-OP QuickDASH, mean±SD |
| 0.086 | 0.0±0.3 | 0.2±0.8 | Pre-OP pain, mean±SD |
| 0.012* | 0 | 15 (14.2) | Lymphedema, n (%) |
| 0.029* | 1 (2.2.) | 15 (13.9) | AWS, n (%) |

The table describes risk factors for any arm morbidity (functional limitation using QuickDASH, pain using numeric pain rating scale, and reduction in abduction and flexion ranges of motion) 6 months after breast surgery. Categorical variables are presented as number and percentage and continuous variables are presented as mean and standard deviation (SD). *Significant p-value (<0.05).

*Abbreviations*: **ROM**- Range of motion, **T**.- Treatment, **IORT**- Intraoperative radiation therapy, **Pre-OP**- Preoperative, **AWS**- Axillar web syndrome.
