# Supplementary material for: A comprehensive approach to risk factors for upper arm morbidities following breast cancer treatment: a prospective study
Source: BMC Cancer. 2021 Nov 20;21:1251. doi: 10.1186/s12885-021-08891-5 (PMC8605604; doi:10.1186/s12885-021-08891-5)
Supplement: Supplementary file 2 — Additional file 2: Table 6. Crosstab and OR divide by two age groups. [file 12885_2021_8891_MOESM2_ESM.docx]

**Tables 6.** Crosstab and OR divide by two age groups.

| **95% CI** | **OR** | **p-value** | **Age >50** | **Age <50** | **Variable** |
| --- | --- | --- | --- | --- | --- |
| 0.61-12.2 | 3.05 | 0.153 | 7 (8.1) | 2 (2.8) | Function disabilities N (%) |
| 0.30-1.12 | 0.58 | 0.107 | 30 (37.5) | 34 (50.7) | Pain N (%) |
| 0.40-1.48 | 0.776 | 0.444 | 30 (34.9) | 29 (40.8) | Decrease ROM N (%) |

*Abbreviations*: **N**-number, **Rom**- Ranfe of motion
