# Supplementary material for: A comprehensive approach to risk factors for upper arm morbidities following breast cancer treatment: a prospective study
Source: BMC Cancer. 2021 Nov 20;21:1251. doi: 10.1186/s12885-021-08891-5 (PMC8605604; doi:10.1186/s12885-021-08891-5)
Supplement: Supplementary file 3 — Additional file 3: Table 7. Crosstab and OR divide by two BMI groups. [file 12885_2021_8891_MOESM3_ESM.docx]

**Tables 7.** Crosstab and OR divide by two BMI groups.

| **95% CI** | **OR** | **p-value** | **BMI>30** | **BMI <30** | **Variable** |
| --- | --- | --- | --- | --- | --- |
| 0.10-7.17 | 0.84 | 0.880 | 1 (5.0) | 8 (5.8) | Function disabilities N (%) |
| 0.32-1.50 | 0.89 | 0.835 | 7 (41.2) | 57 (43.8) | Pain N (%) |
| 0.24-1.87 | 0.67 | 0.454 | 6 (30.0) | 53 (38.7) | Decrease ROM N (%) |

*Abbreviations*: **OR**: Adjusted odds ratio, **CI**: Confidence interval, **BMI**: Body mass index **N**- Number, **ROM**- Range of motion
