# Supplementary material for: A comprehensive approach to risk factors for upper arm morbidities following breast cancer treatment: a prospective study
Source: BMC Cancer. 2021 Nov 20;21:1251. doi: 10.1186/s12885-021-08891-5 (PMC8605604; doi:10.1186/s12885-021-08891-5)
Supplement: Supplementary file 4 — Additional file 4: Table 8. Crosstab and OR divide by the mean amount in the drains. [file 12885_2021_8891_MOESM4_ESM.docx]

**Tables 8.** Crosstab and OR divide by the mean amount in the drains.

| **95% CI** | **OR** | **p-value** | **Mean drain daily amount (cc) >20** | **Mean drain daily amount (cc) <20** | **Variable** |
| --- | --- | --- | --- | --- | --- |
| 1.27-5.78 | 2.71 | 0.009* | 23 (59.0) | 35 (34.7) | Function disabilities N (%) |
| 0.63-2.69 | 1.30 | 0.467 | 18 (41.9) | 38 (35.5) | Pain N (%) |
| 0.15-4.23 | 0.82 | 0.814 | 2 (4.7) | 6 (5.6) | Decrease ROM N (%) |

*Abbreviations*: **OR**: Adjusted odds ratio, **CI**: Confidence interval, **N**- Number, **ROM**- Range of motion
