# Supplementary material for: A comprehensive approach to risk factors for upper arm morbidities following breast cancer treatment: a prospective study
Source: BMC Cancer. 2021 Nov 20;21:1251. doi: 10.1186/s12885-021-08891-5 (PMC8605604; doi:10.1186/s12885-021-08891-5)
Supplement: Supplementary file 5 — Additional file 5: Table 9. Crosstab and OR divide by the size of the tumor. [file 12885_2021_8891_MOESM5_ESM.docx]

**Tables 9.** Crosstab and OR divide by the size of the tumor.

| **95% CI** | **OR** | **p-value** | **Tumor size (cm) >1.1** | **Tumor size (cm) <1** | **Variable** |
| --- | --- | --- | --- | --- | --- |
| 0.73-12.78 | 3.07 | 0.107 | 6 (9.7) | 3 (3.4) | Function disabilities N (%) |
| 0.48-1.87 | 0.95 | 0.895 | 26 (43.3) | 36 (44.4) | Pain N (%) |
| 0.74-2.80 | 1.44 | 0.279 | 27 (43.5) | 31 (34.8) | Decrease ROM N (%) |

*Abbreviations*: **OR**: Adjusted odds ratio, **CI**: Confidence interval, **N**- Number, **ROM**- Range of motion
