# Supplementary material for: A comprehensive approach to risk factors for upper arm morbidities following breast cancer treatment: a prospective study
Source: BMC Cancer. 2021 Nov 20;21:1251. doi: 10.1186/s12885-021-08891-5 (PMC8605604; doi:10.1186/s12885-021-08891-5)
Supplement: Supplementary file 6 — Additional file 6: Table 10. Crosstab and OR divide by the number of dissected lymph nodes. [file 12885_2021_8891_MOESM6_ESM.docx]

**Tables 10.** Crosstab and OR divide by the number of dissected lymph nodes.

| **95% CI** | **OR** | **p-value** | **Lymph node >3** | **Lymph node<3** | **Variable** |
| --- | --- | --- | --- | --- | --- |
| 0.84- 20.91 | 4.20 | 0.060 | 7 (9.7) | 2 (2.5) | Function disabilities n (%) |
| 1.01-3.84 | 1.97 | 0.044* | 33 (45.8) | 24 (30.0) | Pain n (%) |
| 1.44-5.71 | 2.87 | 0.002* | 40 (56.3) | 22 (31.0) | Decrease ROM n (%) |

*Abbreviations*: **OR**: Adjusted odds ratio, **CI**: Confidence interval, **N**- Number, **ROM**- Range of motion
