# Supplementary material for: A comprehensive approach to risk factors for upper arm morbidities following breast cancer treatment: a prospective study
Source: BMC Cancer. 2021 Nov 20;21:1251. doi: 10.1186/s12885-021-08891-5 (PMC8605604; doi:10.1186/s12885-021-08891-5)
Supplement: Supplementary file 7 — Additional file 7: Table 11. Crosstab and OR divide by the mean pain reported during hospitalization. [file 12885_2021_8891_MOESM7_ESM.docx]

**Tables 11.** Crosstab and OR divide by the mean pain reported during hospitalization.

| **95% CI** | **OR** | **p-value** | **Mean hospital pain >0.6** | **Mean hospital pain <0.5** | **Variable** |
| --- | --- | --- | --- | --- | --- |
| 1.00-3.79 | 1.95 | 0.047* | 28 (47.5) | 31 (31.6) | Function disabilities N (%) |
| 1.66-6.71 | 3.34 | *0.001 | 34 (61.8) | 30 (32.6) | Pain N (%) |
| 1.29-32.23 | 6.46 | 0.010* | 7 (11.9) | 2 (2.0) | Decrease ROM N (%) |

*Abbreviations*- **OR**: Adjusted odds ratio, **CI**: Confidence interval, **ROM**: Range of motion,
